# Wavy‐Interlocked Stretchable Triboelectric Nanogenerators Enhanced by Liquid Metal Microflowers for Self‐Powered Wearable Motion Monitoring

**DOI:** 10.1002/advs.202521311

**Published:** 2026-02-12

**Authors:** Qianqian Xu, Huimin Li, Hanmin Zeng, Yahui Meng, Yimeng He, Xinke Hu, Senfeng Zhao, Jianxun Zhang, Peiqiong Zhou, Kechao Zhou, Dou Zhang, Chris Bowen, Yan Zhang

**Affiliations:** ^1^ State Key Laboratory of Powder Metallurgy Central South University Changsha Hunan China; ^2^ Department of Mechanical and Aerospace Engineering Hong Kong University of Science and Technology Hong Kong SAR China; ^3^ Hunan Provincial Key Laboratory of Micro & Nano Materials Interface Science College of Chemistry and Chemical Engineering Central South University Changsha Hunan China; ^4^ Department of Mechanical Engineering University of Bath Bath UK

**Keywords:** liquid metal, motion monitoring, self‐powered sensor, triboelectric nanogenerators

## Abstract

The increasing demand for sustainable, stretchable power sources in wearable electronics has driven the development of high‐performance triboelectric nanogenerators (TENGs). Despite significant progress, stretchable TENGs continue to encounter critical challenges, including insufficient charge density, unstable output performance, and degraded durability when subject to large levels of deformation, which hinder their integration into high‐performance flexible wearable systems. In this work, we construct a stretchable TENG by fabricating an interlocking wavy architecture using electrically conductive eutectic gallium‐indium microflowers (EGaIn MFs) embedded into optimized dielectric membranes based on poly(vinylidene fluoride‐trifluoroethylene) (PVDF‐TrFE) and Nylon‐6 nanofibers. The multi‐petaled, wrinkled microflower morphology of the resulting structure is shown to increase the specific surface area and hierarchical roughness. The electron‐rich properties of the EGaIn MFs promote the formation of the β‐phase in PVDF‐TrFE and the γ‐phase in Nylon‐6, and also act as localized electrically conductive “islands” which reduce the equivalent dielectric thickness and increase the overall capacitance. Based on using membranes with an optimized EGaIn MF loading level, the triboelectric layers were patterned into an interlocked wavy architecture, which expanded the effective contact area and provided a high level of stretchability. The fabricated stretchable TENG with a 30° corrugation angle exhibited a 60% tensile strain, a peak output of 188.6 V and 31.1 µA, and a sensitivity of 3.38 V per unit strain and 0.51 µA per unit strain, respectively. Finally, the device was successfully used for real‐time gesture recognition, demonstrating its potential for self‐powered wearable sensing and intelligent human‐machine interfaces.

## Graphical abstract

Schematic showing a stretchable triboelectric nanogenerator based on interlocked wavy architectures that uses EGaIn microflower‐embedded PVDF‐TrFE and Nylon‐6 nanofiber membranes. The multi‐petaled, electron‐rich EGaIn microflowers enhance interfacial polarization and capacitance, while the interlocked wavy architecture enlarges the effective contact area and accommodates large tensile deformation, enabling a high stretchability of up to 60%, while maintaining a peak output of 188.6 V and 31.1 µA. This new TENG architecture was successfully employed for real‐time wearable motion sensing and gesture recognition, thereby demonstrating its significant potential in applications related to flexible electronics.

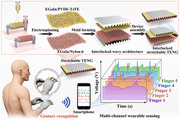

## 1 Introduction

The rapid development of wearable electronics, biomedical monitoring systems, and wireless sensor networks has placed increasing demands on sustainable, flexible energy harvesting and sensing technologies [1, 2, 3]. Triboelectric nanogenerators (TENGs), which exploit the synergistic effect of triboelectrification and electrostatic induction, are able to efficiently convert mechanical stimuli into electrical energy [4]. As a result of their low cost, broad material availability, and environmental friendliness, TENGs exhibit unique advantages in low‐frequency mechanical energy harvesting [5], in particular in the area of self‐powered signal sensing [6]. Among the range of potential device architectures, the formation of a stretchable TENG that can adapt its shape to soft human skin provides new opportunities for next‐generation wearable electronics [7], personal healthcare [8], and human‐machine interfaces [9]. However, their practical application in high‐power wearable devices continues to be limited by their insufficient charge density, unstable output performance, and degraded durability when subject to large levels of strain.

The incorporation of diverse organic or inorganic micro/nano‐scale fillers into a polymer matrix is considered an effective approach for fabricating high‐performance TENGs [10]. For example, He et al. [11] fabricated flexible TENGs by incorporating metallic Ag‐coated perovskite barium zirconium titanate (BZT) nanoparticles as a high‐dielectric filler into polyvinylidene fluoride‐co‐hexafluoropropylene (PVDF‐HFP) polymer fibers, ultimately achieving a 6‐fold increase in the power output. While such approaches provide improvements in the resulting triboelectric output, the significant elastic modulus mismatch between the rigid inorganic fillers and compliant polymer matrices hinders the simultaneous achievement of high electrical output and long‐term stability when subjected to large strains and complex mechanical stresses.

Conductive materials such as reduced graphene oxide, MXene, and Ag nanowires are widely reported to act as charge trapping sites that facilitate interfacial charge accumulation, thereby enhancing the output performance of triboelectric nanogenerators [12, 13]. However, these materials are intrinsically rigid solids with high elastic moduli, which often lead to interfacial stress concentrations, crack initiation, and performance degradation when subject to large deformation levels or repeated mechanical cycling. Liquid metals [14, 15], such as eutectic gallium‐indium (EGaIn) [16], exhibit exceptional electrical conductivity and chemical stability and have emerged as a promising candidate for enhancing the properties of flexible triboelectric systems [17]. Their intrinsic fluidity offers a route for improved elastic modulus matching with highly compliant polymer matrices, thereby effectively reducing interfacial stress concentrations during deformation [18]. However, their high surface tension (≈ 718 mN m^−1^) [19] presents a significant challenge in terms of processing and molding, and their intrinsic fluidic behavior can also introduce the risk of material leakage and a resulting short‐circuit [20].

In addition, conventional stretchable TENGs based on planar architecture are susceptible to interfacial wear and mechanical fatigue when subject to large, repeated levels of deformation, which results in rapid degradation of output performance and a significant reduction in device lifetime. A variety of structural optimization strategies have been proposed to overcome this challenge [21, 22], such as operating in a non‐contact freestanding mode with reduced electrode spacing, the use of rolling‐friction designs to improve stress redistribution, and soft‐contact configurations that employ brush‐like or compliant dielectric interfaces [23]. While these approaches reduce interfacial wear [24], the lack of sufficient interfacial contact inevitably impairs the output performance of the devices.

**FIGURE 1:**
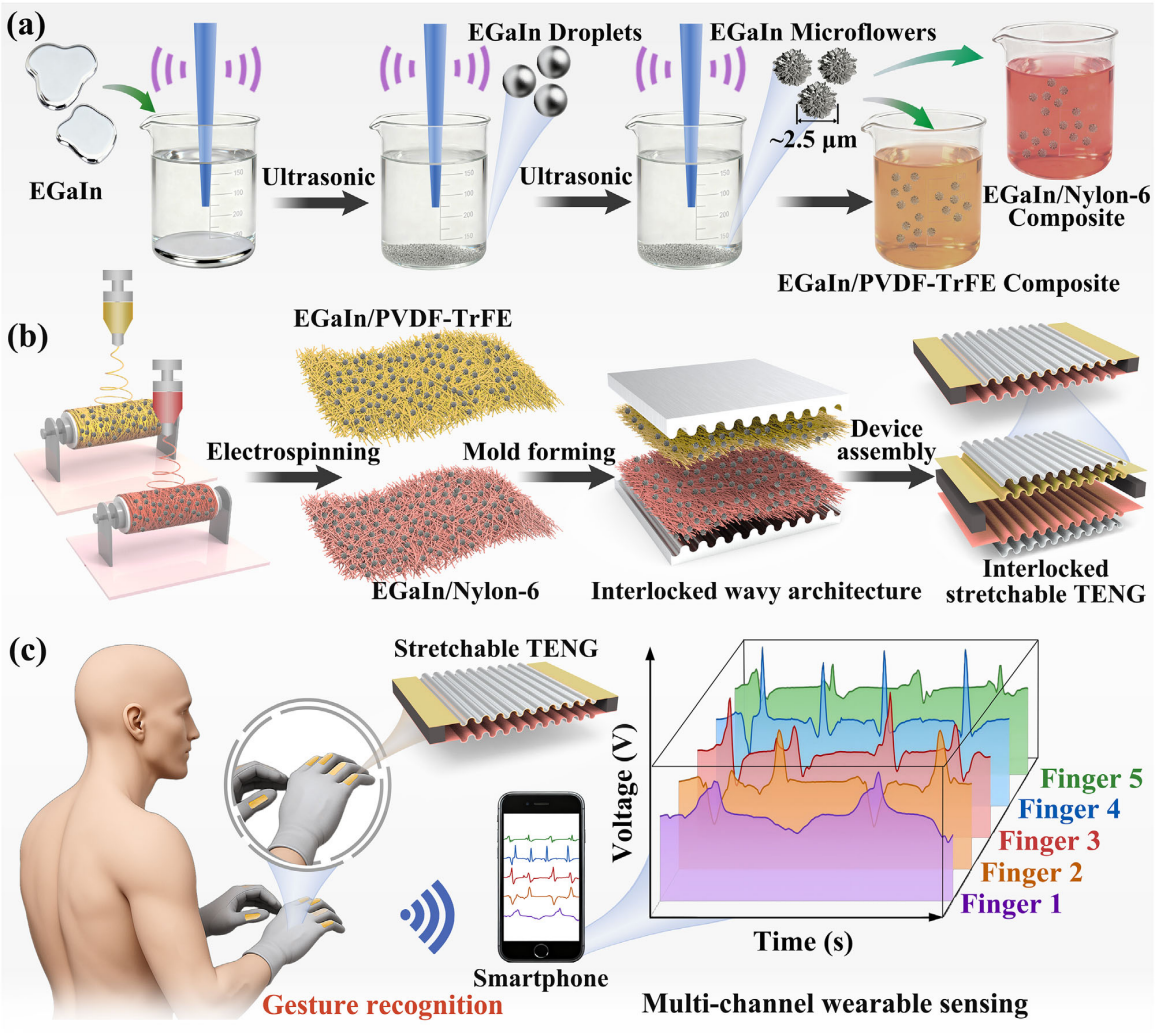
Schematics of the fabrication process for (a) eutectic gallium‐indium microflowers (EGaIn MFs) and (b) wavy‐interlocked stretchable triboelectric nanogenerators (TENGs). (c) Application of the stretchable TENGs as a multi‐channel wearable sensing system for real‐time gesture recognition.

To address these challenges, we propose the development of a stretchable triboelectric nanogenerator that integrates an optimum fraction of EGaIn microflowers (MFs) within a PVDF‐TrFE or Nylon‐6 nanofibrous membrane, which are subsequently formed into an interlocked wavy topology to provide a synergistic route to achieve a high output and reliability during stretching at high levels of strain (see Figure 1). The EGaIn MFs effectively promote the formation of the polar β‐phase in PVDF‐TrFE and the γ‐phase transition of Nylon‐6, thereby significantly enhancing the triboelectric charge trapping and storage capacity. Moreover, within the PVDF‐TrFE/Nylon‐6 nanofibrous network, the EGaIn microflowers act to form localized electrically conductive “islands”, thereby effectively acting as microscale conductive domains which are embedded within the polymeric dielectric layer; this structure acts to reduce the equivalent dielectric thickness and increase the overall capacitance so that the optimized device achieves peak voltage and current outputs of 537.2 V and 133.7 µA, corresponding to 83% and 125% enhancements over the unmodified TENG. To retain the enhancement induced due to the EGaIn MFs, while also imparting flexibility and stretchability, the triboelectric layers based on 6 wt.% EGaIn/PVDF‐TrFE and EGaIn/Nylon‐6 nanofiber membranes were further patterned into a wavy corrugation, thereby forming an interlocked geometry with an enlarged effective contact area. The interlocked device with the 30° wave angle achieved a maximum tensile strain of 60% while generating an open‐circuit voltage (*V*_oc_) and a short‐circuit current (*I*_sc_) of up to 188.6 V and 31.1 µA. The device structure also exhibited significantly improved self‐powered sensing performance, providing voltage and current sensitivities of 3.38 V per unit strain and 0.51 µA per unit strain, which are 196% and 122% greater than the unoptimized device, respectively. Finally, the device was employed to monitor a diverse range of joint movements and successfully enabled real‐time gesture recognition, further demonstrating its significant potential for self‐powered wearable sensing and advanced human‐machine interaction systems.

## 2 Results and Discussion

### 2.1 Material Characteristics

**FIGURE 2:**
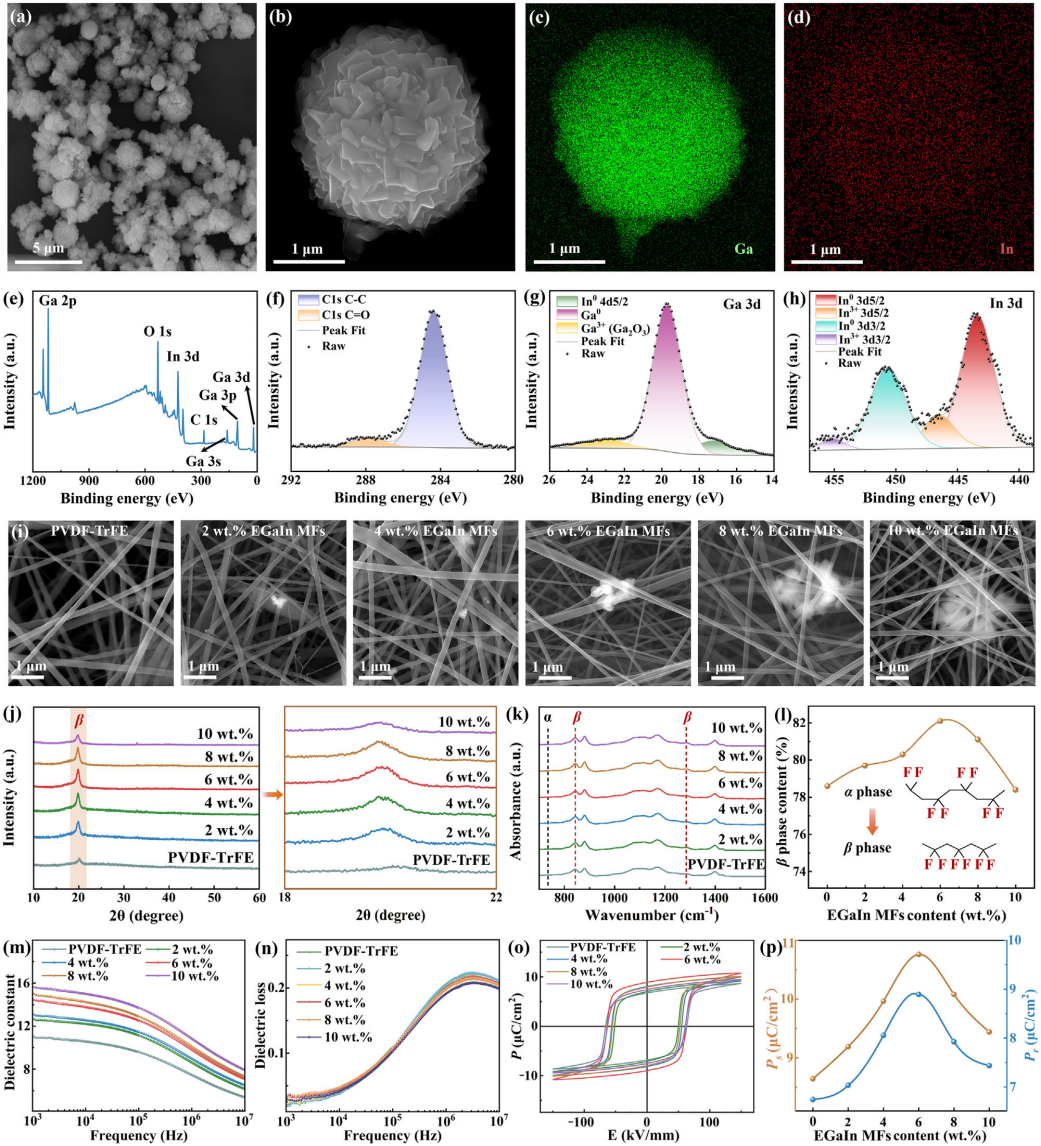
(a) Low‐magnification and (b) single‐particle SEM images of EGaIn microflowers (MFs). (c) EDS mapping image of Ga and (d) In. (e) XPS spectra of EGaIn MFs. High‐resolution XPS spectra of (f) C 1s, (g) Ga 3d, and (h) In 3d. (i) SEM images and (j) XRD analysis results of EGaIn/PVDF‐TrFE nanofiber membranes with varying EGaIn MF contents. (k) FTIR spectra and (l) calculated *β*‐phase fractions of EGaIn/PVDF‐TrFE nanofibers membrane as a function of EGaIn MFs content. (m) Relative permittivity and (n) dielectric loss of EGaIn/PVDF‐TrFE nanofiber membrane. (o) Polarization‐electric field (P‐E) hysteresis loop and (p) corresponding remnant polarization (*P_r_*) and saturation polarization (*P_s_*) of EGaIn/PVDF‐TrFE nanofiber membrane for different EGaIn MF loading levels.

The formation of eutectic gallium‐indium microflowers (EGaIn MFs) is a multi‐step process. Since the surface tension of EGaIn is several tens of times higher than that of water, it will naturally form a droplet‐like shape. Initially, cavitation and shear flow as a result of ultrasonication act to break up the EGaIn droplets; however, the strong surface tension of the alloy forces the droplets to revert to a spherical shape with minimal surface energy. However, with the continued application of high‐energy ultrasound, the fraction of oxide in the alloy layer gradually increases. This increase in rigid oxide acts to inhibit the free shrinkage of the EGaIn droplet, eventually forming localised wrinkles. Finally, the morphology of EGaIn slowly shifts from a smooth spherical shape to a flower‐like structure that is characterized by multi‐petaled wrinkles (see Figure 2a,b). The 3D flower cluster provides a higher specific surface area and surface roughness compared to a spherical geometry, which is particularly beneficial for the generation of interfacial triboelectric charge and enhances the stability of bonding with the polymer matrix. Figure 2c,d presents the energy‐dispersive X‐ray spectroscopy (EDS) mapping of the EGaIn microflowers (MFs), revealing a uniform distribution of Ga and In elements. To further identify the valence states of the constituent elements, X‐ray photoelectron spectroscopy (XPS) analysis was performed; see Figure 2e. The characteristic peak of C 1s may be related to environmentally exposed or surface‐adsorbed carbon species (Figure 2f). Figure 2g,h shows the high‐resolution XPS spectrum of Ga 3d and In 3d. The characteristic peak of Ga 3d exhibits a dominant peak at 19.78 eV, corresponding to the metal Ga^0^, and a weaker peak at 23.18 eV, corresponding to Ga^3+^ ions, mainly from Ga_2_O_3_ [25]. The characteristic peaks of In 3d can be attributed to metal In^0^ (443.58, 451.18 eV) and In^3+^ ions (446.88, 454.98 eV). These results indicate that EGaIn MFs are primarily metallic in nature, with a thin layer of Ga_2_O_3_.

To construct high‐performance triboelectric devices, EGaIn MFs were uniformly dispersed in a PVDF‐TrFE solution, and electrospinning was then used to prepare an EGaIn/PVDF‐TrFE nanofiber membrane. Compared to traditional casting approaches, electrospinning enables the formation of nanofibers with a uniform aspect ratio and a porous network structure, providing the composite membrane with a high level of mechanical flexibility and an increased specific surface area [26]. SEM images show that the EGaIn MFs are uniformly embedded in the PVDF‐TrFE porous fiber network; see Figure 2i. The crystalline phase evolution of the EGaIn/PVDF‐TrFE nanofiber membrane was systematically investigated. As shown in Figure 2j, X‐ray diffraction (XRD) analysis revealed that the characteristic peak intensity of the polar β‐phase of PVDF‐TrFE increased with EGaIn MF content up to 6 wt.% and subsequently decreased. A similar trend was observed in the Fourier‐transform infrared spectroscopy (FTIR) spectra, where the vibrational peaks at 843 and 1280 cm^−1^ reach a maximum intensity at an EGaIn MF content of 6 wt.%, corresponding to an increase in the polar β‐phase content from 78.6% to 82.1%; see Figure 2k,l. With a further increase in EGaIn MF content, the β‐phase content of PVDF‐TrFE decreased to 78.4%. These results indicate that the incorporation of an appropriate amount of EGaIn MFs favors the formation of a polar β‐phase. This enhancement is primarily attributed to the electron‐rich nature of the EGaIn MFs, which interacts electrostatically with the positively charged ‐CH_2_ groups in the PVDF‐TrFE chains, thereby aligning them in the same orientation and facilitating the β‐phase transformation in PVDF‐TrFE. However, at higher EGaIn MF contents, agglomeration of the microflowers becomes significant, which in turn inhibits the β‐phase transition. As shown in Figure 2m and Figure S1, the dielectric constant at 1 kHz increases from 11.0 to 15.6 with increasing EGaIn MFs content, and this is primarily a result of the accumulation of space charges and an enhancement in the level of interfacial polarization at the interface between the EGaIn MFs and the PVDF‐TrFE matrix, thereby enhancing the charge storage capacity. The dielectric loss increases with increasing frequency in the low‐frequency region but decreases at higher frequencies due to dipole relaxation; see Figure 2n. As shown in Figure 2o, all EGaIn/PVDF‐TrFE nanofiber membranes exhibit square‐like and symmetrical ferroelectric hysteresis loops, confirming that the ferroelectric response is well preserved due the formation of the polar β‐phase in PVDF‐TrFE after the addition of EGaIn MFs. Moreover, the saturation polarization (*P_s_*) and remnant polarization (*P_r_*) initially increase with increasing EGaIn MF content, reaching maximum values of 10.8 and 8.9 µC cm^−2^ at 6 wt.%, respectively; see Figure 2p. As the EGaIn MF content further increases to 10 wt.%, the level of dielectric loss increases, and thus *P_s_* and *P_r_* decrease to 9.4 and 7.4 µC cm^−2^, respectively. As shown in Figure S2, the breakdown strength exhibits a similar trend, increasing to 278 kV/mm at 6 wt.% with a 16.8% enhancement compared to pristine PVDF‐TrFE, and then decreasing at higher MF contents. These results indicate that the introduction of an appropriate amount of EGaIn MFs effectively enhances the dielectric and ferroelectric properties of the composite films, although excessive loading levels (> 6 wt.%) lead to increased leakage current and a reduction in breakdown strength.

To fully utilize the high electrical conductivity and interfacial polarization associated with the use of EGaIn MFs, the EGaIn MFs were incorporated into Nylon‐6 nanofibers to fabricate high‐performance triboelectric positive layers; these positive layers are aimed to operate with the negative layers based on EGaIn MFs in PVDF‐TrFE to produce the triboelectric response. As shown in Figure S3, the EGaIn/Nylon‐6 nanofiber membranes with varying EGaIn MF contents exhibit a loosely fibrous network, where the bright regions correspond to EGaIn MFs attached to the Nylon‐6 fibres. Based on optical microscopy observations (Figure S4), the EGaIn MFs are immobilized within the electrospun fibrous networks of both PVDF‐TrFE and Nylon‐6 membranes, and no apparent leakage or exudation is observed. XRD analysis further confirms the successful incorporation of EGaIn MFs; see Figure S5. The diffraction peak at 35.2° is attributed to the characteristic reflection of EGaIn, and the diffraction peak at 21.3° corresponds to the γ‐phase of the Nylon‐6. With increasing EGaIn MF filler content, the γ‐phase diffraction intensity first increases and then decreases at high filler levels, reaching a maximum γ‐phase peak at a filler content of 6 wt.%. Among the crystalline phases, the γ‐phase exhibits a higher net dipole moment and a stronger tendency for charge‐donation [27, 28].

### 2.2 Optimization of EGaIn MF Content and Triboelectric Enhancement Mechanism

**FIGURE 3:**
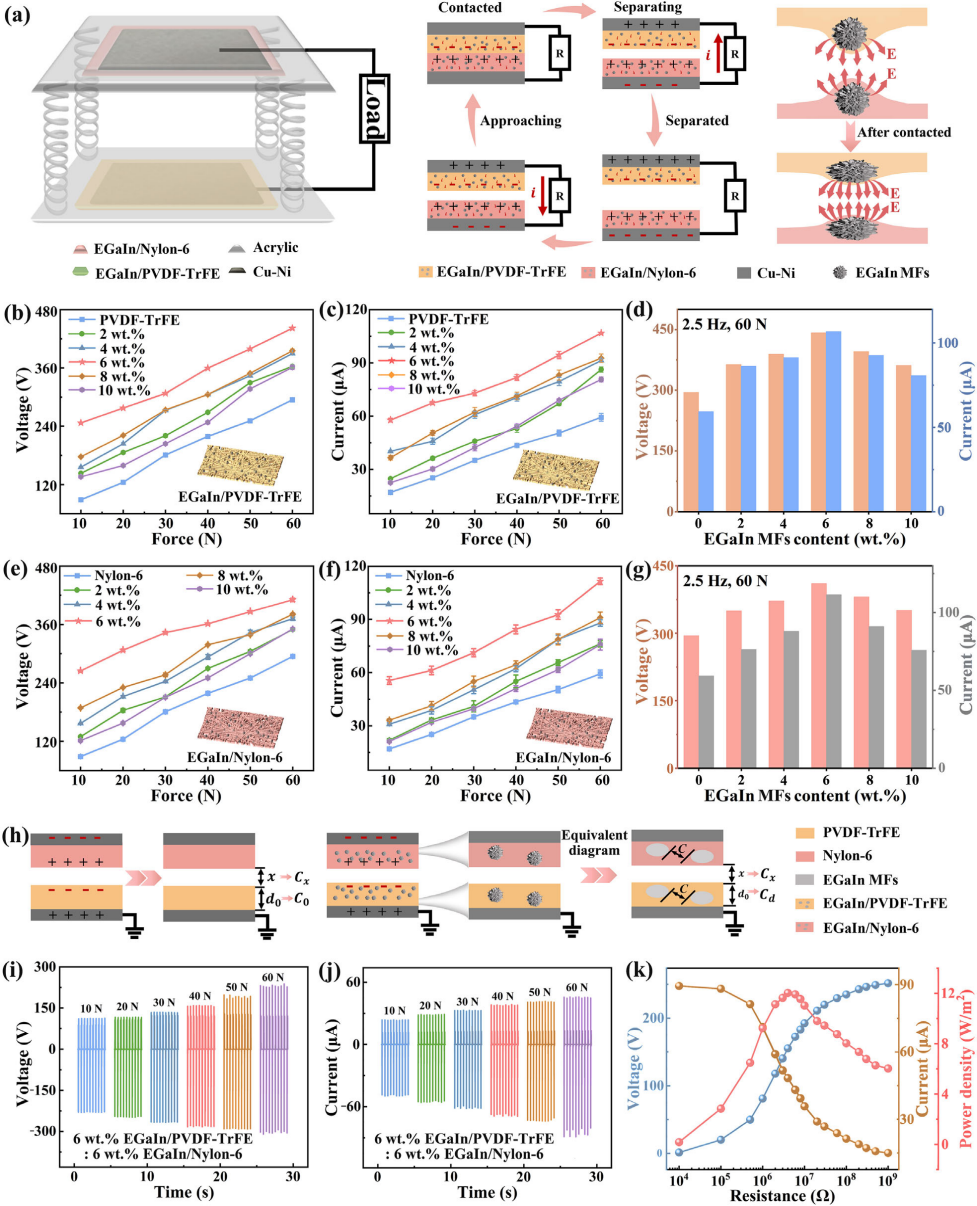
(a) Schematics of the vertical contact‐separation TENG with negative (EGaIn/PVDF‐TrFE) and positive (EGaIn/Nylon‐6) triboelectric layers. (b) Open‐circuit voltage, *V*_oc_, and (c) short‐circuit current, *I*_sc_, of the triboelectric device using EGaIn/PVDF‐TrFE as the negative triboelectric layer and pristine Nylon‐6 as the positive layer under different compression forces. (d) Output performance of TENGs based on EGaIn/PVDF‐TrFE nanofibrous membranes with various EGaIn MFs contents at 60 N. (e) *V*_oc_ and (f) *I*_sc_ of TENGs employing EGaIn/Nylon‐6 nanofibrous membranes as the positive triboelectric layer and pristine PVDF‐TrFE as the negative counterpart under different compression forces. (g) Output performance of TENGs based on EGaIn/Nylon‐6 nanofibrous membranes with different EGaIn MFs contents at 60 N. (h) Schematics of the equivalent capacitance model of TENGs without (left) and with (right) EGaIn MFs incorporation. (i) *V*_oc_ and (j) *I*_sc_ of the optimized TENGs composed of 6 wt.% EGaIn/PVDF‐TrFE and 6 wt.% EGaIn/Nylon‐6 nanofibrous membranes under various external forces. (k) output voltage (peak to peak), output current (peak to peak), and maximum power density of TENGs as a function of load impedance at a force of 60 N.

To investigate the optimal EGaIn MF loading in the two types of nanofiber membranes (PVDF‐TrFE and Nylon‐6), a conventional vertical contact‐separation mode TENG was initially constructed; see Figure 3a and Figure S6. In a typical operation cycle, physical contact between the two triboelectric layers generates opposite triboelectric charges due to their inherent polarity differences. Upon separation, the resulting electric potential difference drives electrons through the external circuit from the bottom electrode to the top electrode. When the maximum separation distance is reached, electrostatic equilibrium is established, and the current flow ceases. As the two layers re‐approach and re‐contact, the balance is disrupted, thereby generating a reverse current. Under repeated mechanical stimulation, this process produces a continuous alternating current output. During periodic contact‐separation events, the relative motion and repeated contact between the EGaIn MFs and the polymer matrices generate additional triboelectric charges at the filler‐polymer interfaces. These charges induce localized electric fields within the triboelectric layers, which can trigger secondary polarization of the conductive EGaIn MFs when subject to mechanical compression or impact [29]. Such secondary polarization facilitates continuous charge redistribution and transfer during cyclic contact‐separation events, contributing to an increased effective surface charge density.

As shown in Figure 3b–d, when 6 wt.% EGaIn was incorporated into the PVDF‐TrFE nanofiber membrane as the negative triboelectric layer with pristine Nylon‐6 as the positive layer, the *V*_oc_ and *I*_sc_ of the resulting TENG increased significantly, from 294.4 V and 59.3 µA to 441.7 V and 106.7 µA at 60 N, respectively. However, further increasing the EGaIn content to 10 wt.% results in a decrease in performance (361.2 V and 80.6 µA). Kelvin probe force microscopy (KPFM) was conducted to measure the surface potential changes of the EGaIn/PVDF‐TrFE nanofiber membrane. As shown in Figure S7, the PVDF‐TrFE membrane exhibited a surface potential of −182.5 mV, while the incorporation of 6 wt.% of EGaIn MFs resulted in a surface potential that significantly shifted to ‐858.9 mV, showing a 371% increase in negativity. This enhancement is attributed to the EGaIn MFs acting as triboelectric charge traps, which lower the Fermi level and facilitate charge transfer. Moreover, the interfacial polarization between the EGaIn MFs and PVDF‐TrFE matrix promotes the formation of a localized electric field. However, when the EGaIn MFs content increased to 10 wt.%, the surface potential shifted back to −366.2 mV; this decrease is likely due to particle aggregation caused by reduced interparticle spacing, which inhibits β‐phase formation in the PVDF‐TrFE and disrupts the unidirectional alignment of ferroelectric dipoles (see Figure S8). These results demonstrate that the 6 wt.% EGaIn/PVDF‐TrFE nanofiber membrane achieves a significant negative shift in surface potential by balancing conductivity and charge trapping ability, thereby significantly improving triboelectric output performance.

A similar trend was observed when EGaIn/Nylon‐6 nanofiber membranes were employed as the positive triboelectric layer, with pristine PVDF‐TrFE as the negative counterpart. As shown in Figure 3e–g, the *V*_oc_ and *I*_sc_ reached peak values of 410.5 V and 111.4 µA at 6 wt.% EGaIn MFs content, increased by approximately 39.4% and 87.9%, respectively. KPFM analysis indicated a corresponding increase in surface potential from 239.8 to 1171.1 mV (a 388% enhancement); this was facilitated by the increased γ‐phase crystallinity and stronger interchain hydrogen bonding promoted by EGaIn MFs; see Figure S9. Accordingly, the surface potential difference between the positive and negative electrode layers reached a peak of 1353.6 mV at 6 wt.% EGaIn MF loading, a 221% increase compared to the pristine configuration (422.3 mV). These results demonstrate that the 6 wt.% EGaIn/Nylon‐6 nanofiber membranes, with their abundant γ‐phase in the Nylon‐6 and optimal surface potential, serve as the most effective positive triboelectric layer for the construction of high‐performance TENGs.

To further understand the role of EGaIn MFs in enhancing the triboelectric output, an equivalent capacitance model was established, as shown in Figure 3h. The embedded EGaIn MFs within the nanofibrous membranes act as localized electrically conductive “islands”, which are analogous to the insertion of ultrathin metallic layers into the dielectric medium of a parallel‐plate capacitor, thereby increasing the total capacitance [30]. The enhanced effective capacitance facilitates an improvement in both charge storage and the transfer efficiency of charge, which is consistent with the experimentally observed improvements in output performance. Although the EGaIn MFs are not continuously distributed, the formation of electrically conductive “islands” significantly enhances the dielectric response by promoting the formation of local field concentrations and interface polarization, thereby effectively enhancing the dielectric response and triboelectric output.

Based on the optimized configuration, a high‐performance TENG was constructed using 6 wt.% EGaIn/PVDF‐TrFE and EGaIn/Nylon‐6 nanofiber membranes as triboelectric pairs. As shown in Figure 3i,j, both the *V*_oc_ and *I*_sc_ increase steadily with applied force, reaching peak values of 537.2 V and 133.7 µA when subject to a force of 60 N. These values are improved by approximately 83% and 125% compared to devices constructed from pristine PVDF‐TrFE and Nylon‐6 nanofiber membranes, respectively. This result demonstrates the synergistic improvement that is achieved by incorporating EGaIn MFs into both triboelectric layers. The optimized TENGs exhibited a maximum power density of 12 W m^−2^ for an external load resistance of 4 MΩ, demonstrating efficient energy harvesting under low‐frequency mechanical stimuli, see Figure 3k. Long‐term mechanical durability of the optimized TENGs was verified through 10 000 contact‐separation cycles at 2.5 Hz and 60 N, during which the *V*_oc_ remained highly stable, with no observable performance degradation or mechanical damage; see Figure S10. These results confirm the mechanical robustness and electrical reliability of the EGaIn‐enhanced TENGs architecture under prolonged dynamic loading.

### 2.3 Interlocked Wavy Architecture for High‐Performance Stretchable TENGs

**FIGURE 4:**
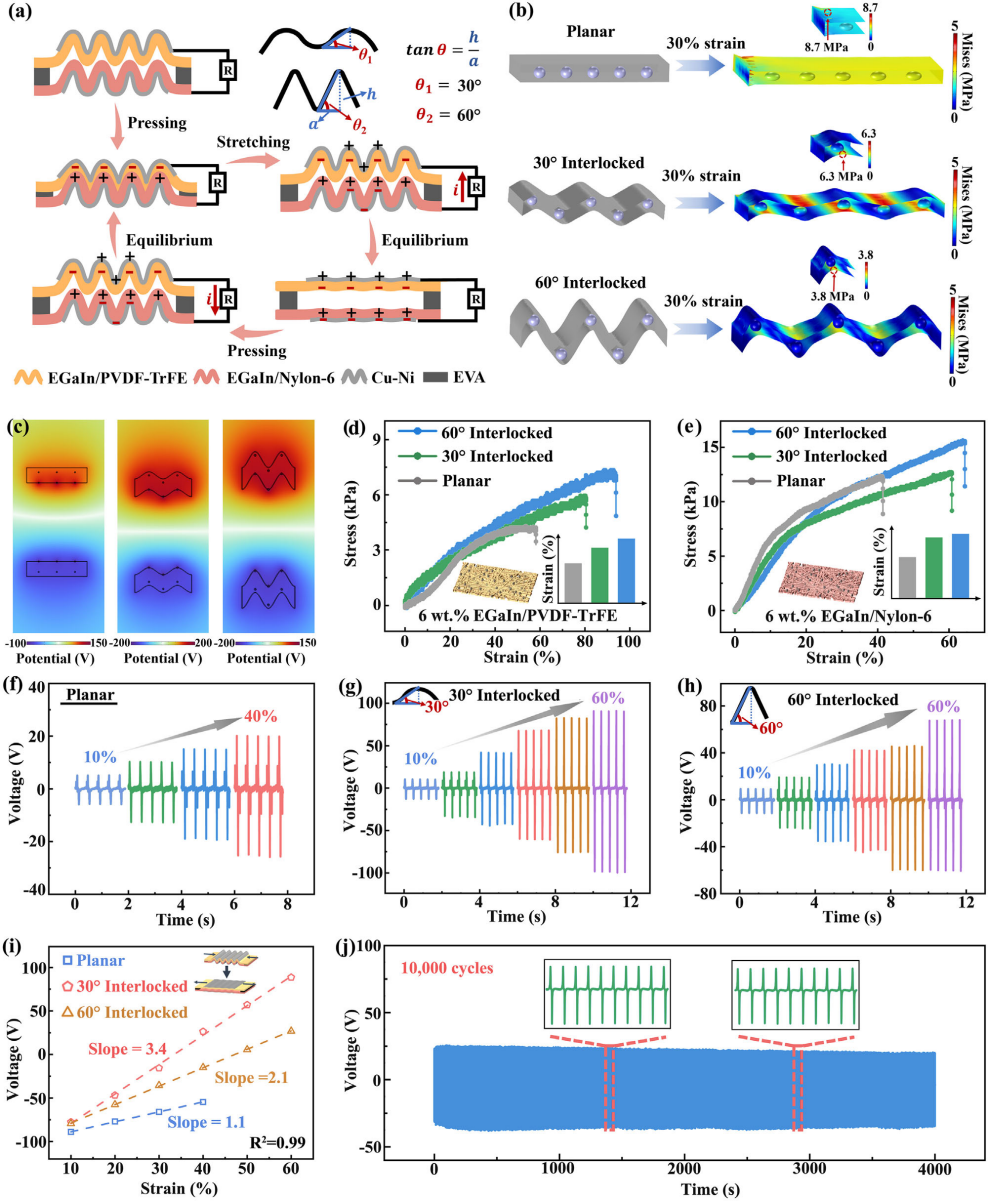
(a) Schematics of the working mechanism of the interlocked stretchable TENGs. Finite element simulation of (b) stress distribution and (c) potential distribution in TENGs with different geometries under 30% tensile strain: (top) planar, (middle) 30° interlocked, and (bottom) 60° interlocked configurations. Stress‐strain curves of (d) 6 wt.% EGaIn/PVDF‐TrFE and (e) 6 wt.% EGaIn/Nylon‐6 nanofibrous membranes with varying corrugation angles. *V*_oc_ and *I*_sc_ of the triboelectric devices with (f) planar, (g) 30° interlocked, and (h) 60° interlocked structures under tensile strains. (i) The voltage‐strain curves of the interlocked stretchable TENGs with different configuration angles. (j) Stretchable TENGs exhibit excellent stability over ≈10,000 cycles under tensile strain of 30% at 2.5 Hz, the inset shows signal details during ten cycles.

To retain the enhancement in output due to the presence of EGaIn MFs, while also achieving a device with flexibility and deformability, a novel stretchable TENG based on an interlocking wavy topology architecture was fabricated. In this device architecture, 6 wt.% EGaIn MFs were first incorporated into both the negative (PVDF‐TrFE) and positive (Nylon‐6) triboelectric layers to enhance the surface charge density. The positive and negative friction layers were then patterned using precision‐milled molds with controlled waviness angles (0°, 30°, and 60°), thereby producing well‐defined sinusoidal profiles. Finally, an elastic ethylene‐vinyl acetate (EVA) foam adhesive replaced the conventional spring component, imparting a high level of resilience to the overall device. The working mechanism of the interlocked TENG differs from the conventional vertical contact‐separation mode, as in Figure 3a, since it relies on horizontal stretching and compression events to induce a periodic contact and separation between the positive and negative triboelectric layers, as shown in Figure 4a. Initially, the upper negative EGaIn/PVDF‐TrFE layer and the lower positive EGaIn/Nylon‐6 layer are completely separated, with electrically neutral surfaces, and the wave‐like peaks of the two layers are offset relative to each other, thereby forming an interlocking arrangement. Under the action of an external force, the upper wave peaks gradually interlock with the lower valleys, establishing local contact and inducing a triboelectric charge. The contact area and accumulated charge reach their maximum values when the peaks are fully embedded in the opposing valleys, corresponding to a maximum level of deformation. Upon stretching, the interlocked units recover, reducing the contact area and driving electrons through an external circuit to balance the potential difference, thereby generating an output current. Residual surface charge remains on the layers, and during subsequent compression, electrostatic induction drives electrons in the opposite direction relative to the initial current, thereby generating an alternating electrical output. Consequently, periodic horizontal compression and stretching lead to the production of a stable alternating current output, whereby the interlocked wave structure enhances the contact area and mechanical robustness.

Finite element simulations were further employed to evaluate the effect of surface topology on the interfacial stress and electrostatic potential; see Figure 4b,c. At an interlayer spacing of 2.6 mm, the planar structure exhibited the highest peak stress of 8.7 MPa. However, due to its confinement to a narrow edge, the resulting potential difference was limited to 289 V. The 30° wavy topology slightly reduced the peak stress to 6.3 MPa, but distributed it more continuously across peaks and valleys, thereby increasing the potential difference to 465 V. When the angle is further increased to 60°, the local stress concentration still exists, however the peak stress decreased to 3.8 MPa, resulting in a decrease in potential to 397 V. These results confirm that maximizing the level of charge generation requires a comprehensive optimizing for both contact area and stress uniformity, not merely pursuing a wavy topology angle.

In addition, surface patterning significantly improved the ductility of the triboelectric membranes, see Figure 4d,e. As the waviness angle increased from 0° to 60°, the elongation to failure of the 6 wt.% nanofiber membrane increased from 58.1% to 92.7%, and the fracture stress increased from 4.2 to 7.3 kPa, respectively. Similarly, EGaIn/Nylon‐6 nanofiber membrane exhibited an enhancement of 57% in fracture elongation and 26% in fracture stress, increasing from 41.0% and 12.4 kPa to 64.4% and 15.6 kPa, respectively.

The interlocked‐structure TENGs were subjected to tensile testing at 2.5 Hz when subject to different levels of strain to evaluate the output performance. As shown in Figures 4f and Figure S11, the *V*_oc_ and *I*_sc_ of all devices gradually increased with tensile strain. Due to the unpatterned device exhibiting a lower elongation to failure, its *V*_oc_ and *I*_sc_ reached peak values of 45.4 V and 8.4 µA at a tensile strain of 40%, respectively. In contrast, the TENGs with a wavy topology exhibited an improved level of stretchability and a wider operational range. The TENGs with a 30° wavy angle attained a peak output of 188.6 V and 31.1 µA at a strain of 60%; see Figures 4g and Figure S12. These enhancements are primarily attributed to the increased effective contact area between the wavy‐architected positive and negative triboelectric layers, while the interlocked architecture promotes the compression and deformation of the EGaIn MFs and facilitates secondary polarization. In comparison, while the 60° device reached a peak output at 60% strain, it delivered a slightly reduced output of 126.9 V and 20.3 µA, see Figures 4h and Figure S13. This result is consistent with the electrostatic potential distribution, indicating that although the 60° topology theoretically offers a larger contact area, the resulting stress distribution reduces interfacial conformity, thereby leading to slightly lower output compared to the 30° structure.

The sensing sensitivities of the interlocked structures were further evaluated when subject to tensile deformation. As shown in Figures 4i and Figure S14, the unpatterned device exhibited the lowest sensor response, with the voltage sensitivity and the current sensitivity of 1.14 V per strain and 0.23 µA per strain, respectively. In contrast, the 30°‐angled interlocked architecture exhibited the highest level of sensitivity, namely 3.38 V per unit strain for voltage and 0.51 µA per unit strain for current, representing approximately 196% and 122% enhancements, respectively, compared with the unpatterned device. Durability testing further confirmed the robust mechanical stability of the interlocked design. After 10 000 continuous stretching cycles at 2.5 Hz, the *V*_oc_ of the 30°‐angled interlocked architecture remained essentially unchanged, exhibiting negligible variation between measurements obtained at the initial and final stages of the test; Figure 4j. After cyclic fatigue testing, no detectable damage was observed in either the composite nanofiber membranes or the assembled devices, demonstrating the robust structural integrity and long‐term operational stability of the interlocked TENGs. Compared with the representative stretchable triboelectric nanogenerators reported in the literature, the proposed stretchable interlocked TENG not only exhibits excellent mechanical properties, but also provides a high electrical output, see Table S1.

### 2.4 Wearable Motion Sensing and Gesture Recognition

Interlocking stretchable TENGs offer unique advantages, including a fast response time, good conformability to human skin, and excellent cycling stability, which provides application potential in the field of biomechanical motion monitoring. Among the range of human joints, finger joints are the most dexterous and finely controlled, which are able to perform multiple‐degrees‐of‐freedom motion such as flexion/extension, abduction/adduction, and complex grasping [31]. These movements not only support basic daily functions but are also closely related to applications that include neurorehabilitation, motion recognition, and human‐computer interaction. Therefore, high‐sensitivity and high‐precision real‐time monitoring of finger movements is crucial for applications ranging from medical rehabilitation to intelligent prosthetics and soft robotics [32].

**FIGURE 5:**
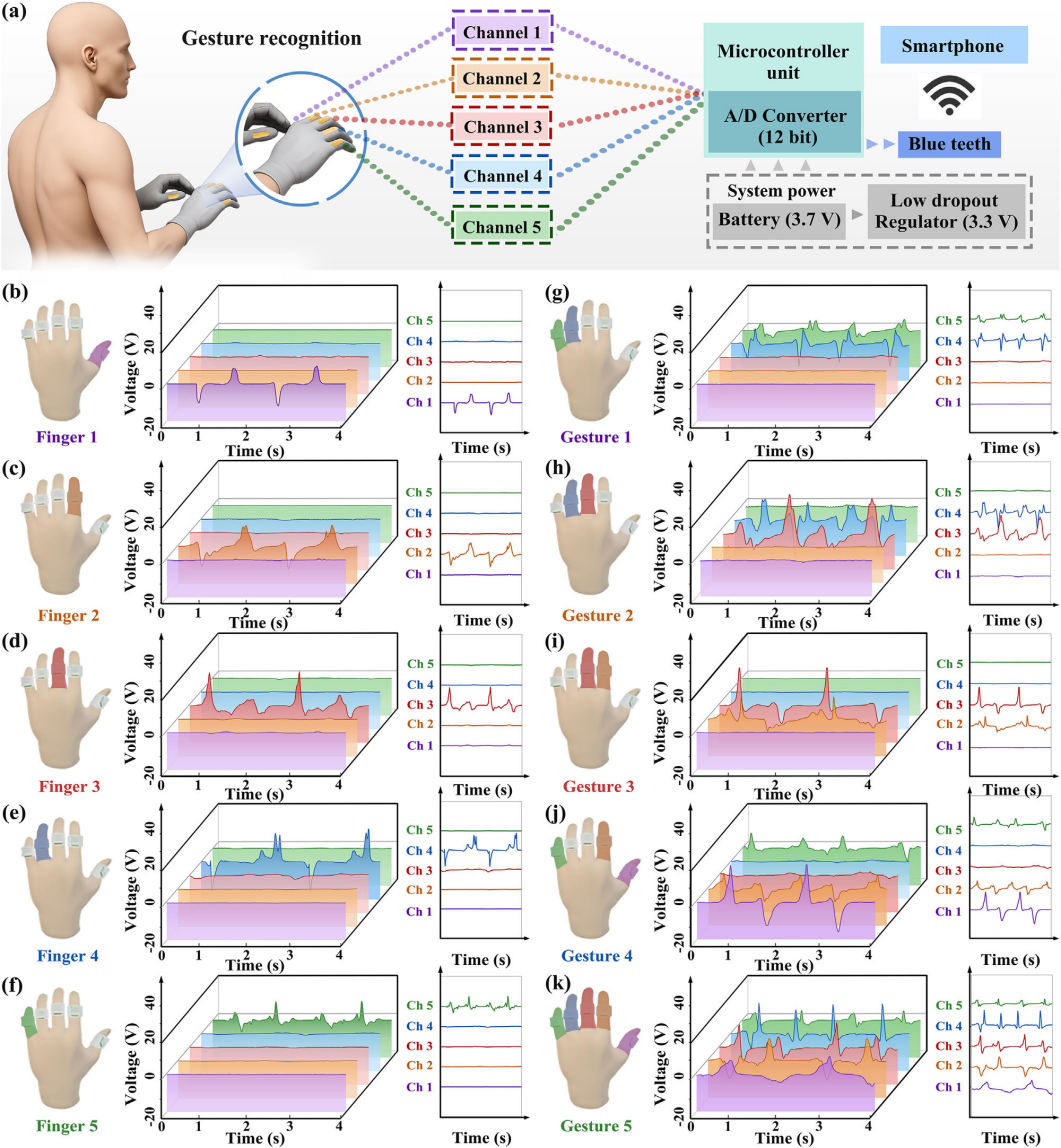
(a) Schematic of a multi‐channel wearable sensing system for real‐time gesture recognition. Five interlocked stretchable TENG units are integrated with a multi‐channel wireless module, and the acquired voltage signals are wirelessly transmitted to a smartphone for real‐time display. (b–f) Output voltage responses corresponding to the bending of single fingers. (g–k) Voltage signals were recorded from each channel during different gesture movements.

To demonstrate the gesture recognition capabilities of an interlocking stretchable TENGs, we developed a multi‐channel wearable sensing system. As shown in Figure 5a, five stretchable triboelectric sensors were attached to individual finger joints and connected to a printed circuit board (PCB) integrated with a multi‐channel wireless transmission module, with each sensor assigned to an independent reading channel. The wireless module consists of a power supply, a 12‐bit A/D converter, a low‐power microcontroller, and a Bluetooth unit. The resulting device transmits voltage signals in real time to a smartphone for intuitive visualization.

As shown in Figure 5b–f and Video S1, the multi‐channel wearable sensing system successfully captured and displayed voltage outputs corresponding to different gestures in real time. When a single finger (thumb, index, middle, ring, or little) was bent, only the corresponding channel generated an obvious voltage signal, while the other channels showed almost negligible signals. This one‐to‐one correspondence between finger motion and voltage output enabled accurate identification of the active finger and its associated gesture. To further evaluate the system's multi‐gesture recognition capability, the responses to simultaneous bending of multiple fingers were analyzed. As shown in Figure 5g–i, when any two fingers were bent simultaneously, two voltage peaks appeared in the corresponding channels. Similarly, bending of any three fingers produced three distinct voltage peaks in their respective channels, clearly validating the system's capability for multi‐finger gesture discrimination; see Figure 5j. During fist clenching, all channels generated clear voltage peaks, confirming both high sensitivity and reliable detection of complex finger movements; see Figure 5k.

In order to further verify the practicality of interlocking stretchable TENGs in wearable motion monitoring, devices were also fixed to the wrist and elbow using EVA (ethylene‐vinyl acetate) double‐sided adhesive. Joint movements induced cyclic stretching and compression of the devices, driving periodic contact‐separation of the triboelectric layers to generate electrical outputs. As shown in Figures S15 and S16, wrist flexion produced an output of ∼13.6 V, while elbow bending and extension generated a voltage of ∼53.7 V due to greater strain and improved interfacial contact. The differences in waveforms and amplitudes observed in these scenarios further demonstrate the application potential of interlocked stretchable TENGs for wearable electronics and real‐time human motion monitoring.

## 3 Conclusion

In conclusion, we have successfully demonstrated stretchable TENGs based on EGaIn microflowers in both negatively charged PVDF‐TrFE and positively charged Nylon‐6 nanofibrous membranes with an interlocked wavy architecture. The application of ultrasonic‐assisted processing was able to reshape smooth EGaIn droplets into multi‐petaled wrinkled microflowers that provided enhanced interfacial polarization and promoted the formation of the polar β‐phase in the PVDF‐TrFE and the γ‐phase transition in Nylon‐6. The EGaIn microflowers also acted to introduce localized electrically conductive “islands” that reduced the effective dielectric thickness and increased the overall capacitance. Consequently, the optimized device achieves peak voltage and current outputs of 537.2 V and 133.7 µA, corresponding to 83% and 125% enhancements over the unmodified TENG, with a maximum power density of 12 W m^−2^. Further patterning of the membranes into an interlocked wavy topology increased the effective contact area, enabling operation at a 60% tensile strain with electrical outputs of 188.6 V and 31.1 µA, and voltage and current sensitivities of 3.38 V and 0.51 µA per unit strain, which are 196% and 122% higher than those of the unpatterned device. The interlocked stretchable TENGs achieved reliable joint motion monitoring and real‐time gesture recognition, revealing significant potential for next‐generation self‐powered wearable sensors and interactive electronics.

## 4 Experimental Section

### 4.1 Preparation of EGaIn/PVDF‐TrFE Nanofiber Membranes

50 mg of polyvinylpyrrolidone was added to 15 mL of a 50 vol.% ethanol‐water solution and sonicated until completely dissolved. Next, 1 g of EGaIn (Cat. no. 495425, Sigma–Aldrich) was added to the polyvinylpyrrolidone solution and pre‐dispersed in an ultrasonic bath for 30 s. The sample was then transferred to a cell disruptor (300 W, 50% duty cycle) and sonicated for 8 hours. During sonication, a cold‐water bath was used to maintain the temperature to approximately 20°C. Upon completion, the EGaIn microflowers (EGaIn MFs) were successfully obtained. After centrifugation, the obtained EGaIn MFs were redispersed in a 13 wt.% PVDF‐TrFE solution in DMF to prepare mixtures containing 2, 4, 6, 8, and 10 wt.% of EGaIn MFs. The mixtures were stirred at 40 °C until the polymer and fillers were fully dissolved and uniformly dispersed. The prepared solutions were subsequently electrospun at 40 °C with a flow rate of 1 mL h^−1^, an applied voltage of 15 kV, and a drum rotation speed of 1000 rev min^−1^. The resulting membranes were then dried at room temperature for 24 h to obtain the EGaIn/PVDF‐TrFE composite membranes.

### 4.2 Fabrication of EGaIn/Nylon‐6 Nanofiber Membranes

A mixed solvent of formic acid and acetic acid in a 1:1 (w/w) ratio was used to dissolve 1.54 g of Nylon‐6 under continuous stirring at 40 °C until a clear and uniform solution was obtained. The resulting solution was electrospun at 40 °C with a flow rate of 1 mL h^−1^, an applied voltage of 20 kV, and a drum rotation speed of 1000 rev min^−1^. The as‐spun fibers were dried at room temperature for 24 h to obtain Nylon‐6 nanofiber membranes. To prepare the EGaIn/Nylon‐6 nanofiber membranes, EGaIn MFs were centrifuged and subsequently dispersed into the Nylon‐6 solution to prepare suspensions with EGaIn MF loadings of 2, 4, 6, 8, and 10 wt.%. The mixtures were stirred at 40 °C until the EGaIn MFs were uniformly dispersed. Electrospinning was then carried out under the same conditions as described above, and the resulting fibers were dried for 24 h to obtain the EGaIn/Nylon‐6 nanofiber membranes.

### 4.3 Design and Construction of EGaIn MF‐Optimized and Interlocked Stretchable TENGs

For the TENGs operating in the vertical contact‐separation mode, the PVDF‐TrFE nanofiber membrane with various EGaIn MF contents and the Nylon‐6 nanofiber membrane with various EGaIn MF loadings were employed as the negative and positive triboelectric layers, respectively. Both membranes were cut into squares with dimensions of 2.5 × 2.5 cm^2^. Copper‐nickel conductive tapes were attached to the back sides of the membranes as electrodes, and copper wires were used to connect the external circuit. The two layers were mounted on acrylic substrates, and a spring‐loaded configuration was adopted to realize periodic contact and separation between the triboelectric layers.

The interlocked flexible TENGs were designed to operate under a lateral stretching‐induced contact and separation mode, and were fabricated using the same 6 wt.% EGaIn/PVDF‐TrFE and 6 wt.% EGaIn/Nylon‐6 nanofiber membranes. Both membranes were patterned using nylon molds with corrugation angles of 30° and 60° to form the sinusoidal interlocked surfaces. Copper‐nickel conductive tapes were applied as electrodes, and the two patterned layers were assembled face‐to‐face using EVA double‐sided adhesive foam as a soft elastic spacer instead of metal springs.

### 4.4 Characterization

X‐ray diffraction (XRD, Malvern Panalytical, UK) was employed to analyze the crystal structure and phase composition of the samples with a scanning speed of 5° min^−1^. The surface morphology and microstructure were observed by field‐emission scanning electron microscopy (FESEM, TESCAN MIRA4, Czech Republic), and the elemental distribution was characterized by energy‐dispersive X‐ray spectroscopy (EDS). Fourier‐transform infrared spectroscopy (FTIR, Thermo Fisher Nicolet IS50, USA) was used to identify the characteristic functional groups and molecular structures of the composite materials with a resolution of 0.4 cm^−1^ over a wavenumber range of 400–3500 cm^−1^. The dielectric properties of the samples were measured using a precision impedance analyzer (4294A, Agilent Technologies, USA). The ferroelectric response was characterized using a ferroelectric analyzer (TF Analyzer 2000, aixACCT Systems, Germany). The surface potential of the positive and negative triboelectric layers was measured by Kelvin probe force microscopy (KPFM, MultiMode 8, Bruker, Germany) using a Pt‐coated silicon probe with an amplitude of 350 mV.

### 4.5 Measurement

The electrical output of the TENGs was evaluated using a linear motor (XF‐DXT178E, LinMot, Switzerland). Rigid devices were subjected to periodic contact‐separation cycles at 2.5 Hz for a range of applied forces, while the stretchable interlocked devices were tested under different tensile strains at the same frequency. The open‐circuit voltage, short‐circuit current, and load power were recorded using a digital multimeter (DMM7510, Keithley, USA).

### 4.6 Finite Element Simulation Analysis

The interlocked triboelectric nanogenerators were modeled using COMSOL Multiphysics 5.4 to simulate the potential distribution during periodic contact‐separation processes. The triboelectric layers were separated by gaps of 0.1 and 2.6 mm, respectively. Wavy surface morphologies with corrugation angles of 0°, 30°, and 60° were constructed to analyze the influence of surface topology on the electrostatic potential distribution.

## Conflicts of Interest

The authors declare no conflict of interest.

## Supporting information




**Supporting File 1**: advs743320‐sup‐0001‐SuppMat.docx.

**Supporting File 2**: advs743320‐sup‐0002‐VideoS1.mp4.

## Data Availability

The data that support the findings of this study are available from the corresponding author upon reasonable request.
