# Supplementary material for: Wavy‐Interlocked Stretchable Triboelectric Nanogenerators Enhanced by Liquid Metal Microflowers for Self‐Powered Wearable Motion Monitoring
Source: Adv Sci (Weinh). 2026 Feb 12:e21311. Online ahead of print. doi: 10.1002/advs.202521311 (PMC13325643; doi:10.1002/advs.202521311)
Supplement: Supplementary file 1 — Supporting File 1: advs743320‐sup‐0001‐SuppMat.docx. [file ADVS-9999-e21311-s001.docx]

Supporting Information

**Wavy-Interlocked Stretchable Triboelectric Nanogenerators Enhanced by Liquid Metal Microflowers for Self‐Powered Wearable Motion Monitoring**

*Qianqian Xu, Huimin Li, Hanmin Zeng, Yahui Meng, Yimeng He, Xinke Hu, Senfeng Zhao, Jianxun Zhang, Peiqiong Zhou,* *Kechao Zhou, Dou Zhang, Chris Bowen*, Yan Zhang**

Q. Xu, H. Li, H. Zeng, Y. Meng, Y. He, X. Hu, J. Zhang, P. Zhou, Prof. K. Zhou, Prof. D. Zhang, Prof. Y. Zhang

State Key Laboratory of Powder Metallurgy, Central South University, Changsha, Hunan, 410000, China.

E-mail: yanzhangcsu@csu.edu.cn

Q. Xu

Department of Mechanical and Aerospace Engineering, Hong Kong University of Science and Technology, Clear Water Bay, Hong Kong SAR, 999077, China.

S. Zhao

Hunan Provincial Key Laboratory of Micro & Nano Materials Interface Science, College of Chemistry and Chemical Engineering, Central South University, Changsha, Hunan, 410083, China.

Prof. C. Bowen

Department of Mechanical Engineering, University of Bath, Bath, BA2 7AY, UK.

E-mail: c.r.bowen@bath.ac.uk

**This PDF file includes:**

Figures S1 to S16 and Table S1.

**Other Supporting Online Information for this manuscript**

Video S1

**Supplementary figures**


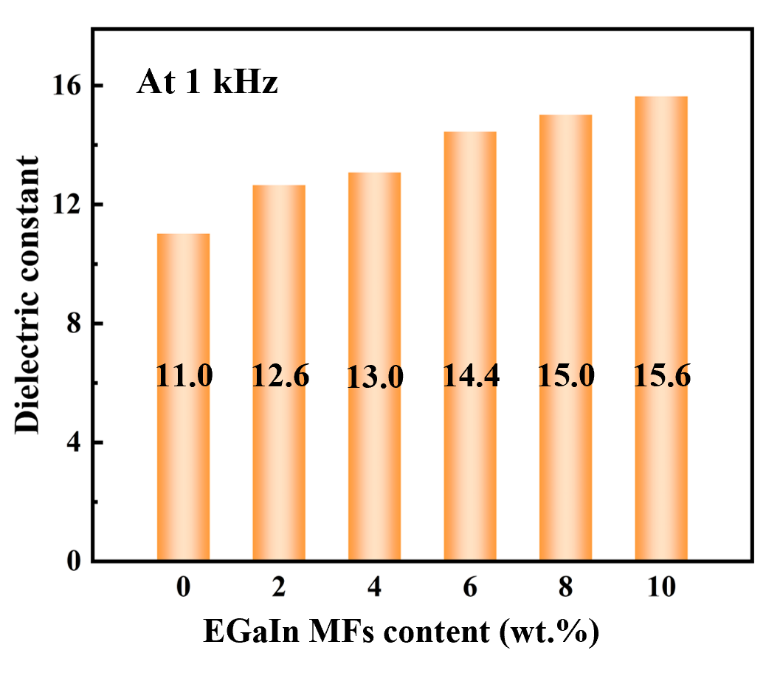


**Figure S1.** Dielectric constant at 1 kHz of PVDF-TrFE-based nanofiber membranes with a range of EGaIn micro-flower (MF) contents.


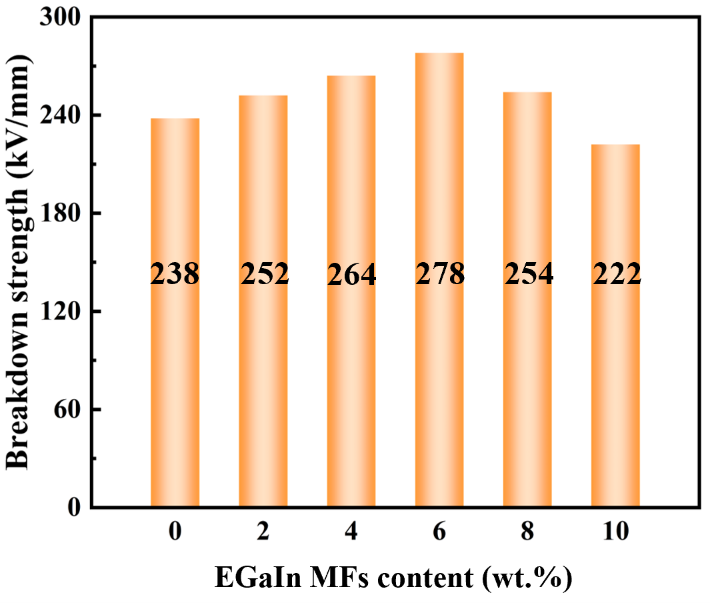


**Figure S2.** Breakdown strength of PVDF-TrFE-based nanofiber membranes with a range of EGaIn MF contents.


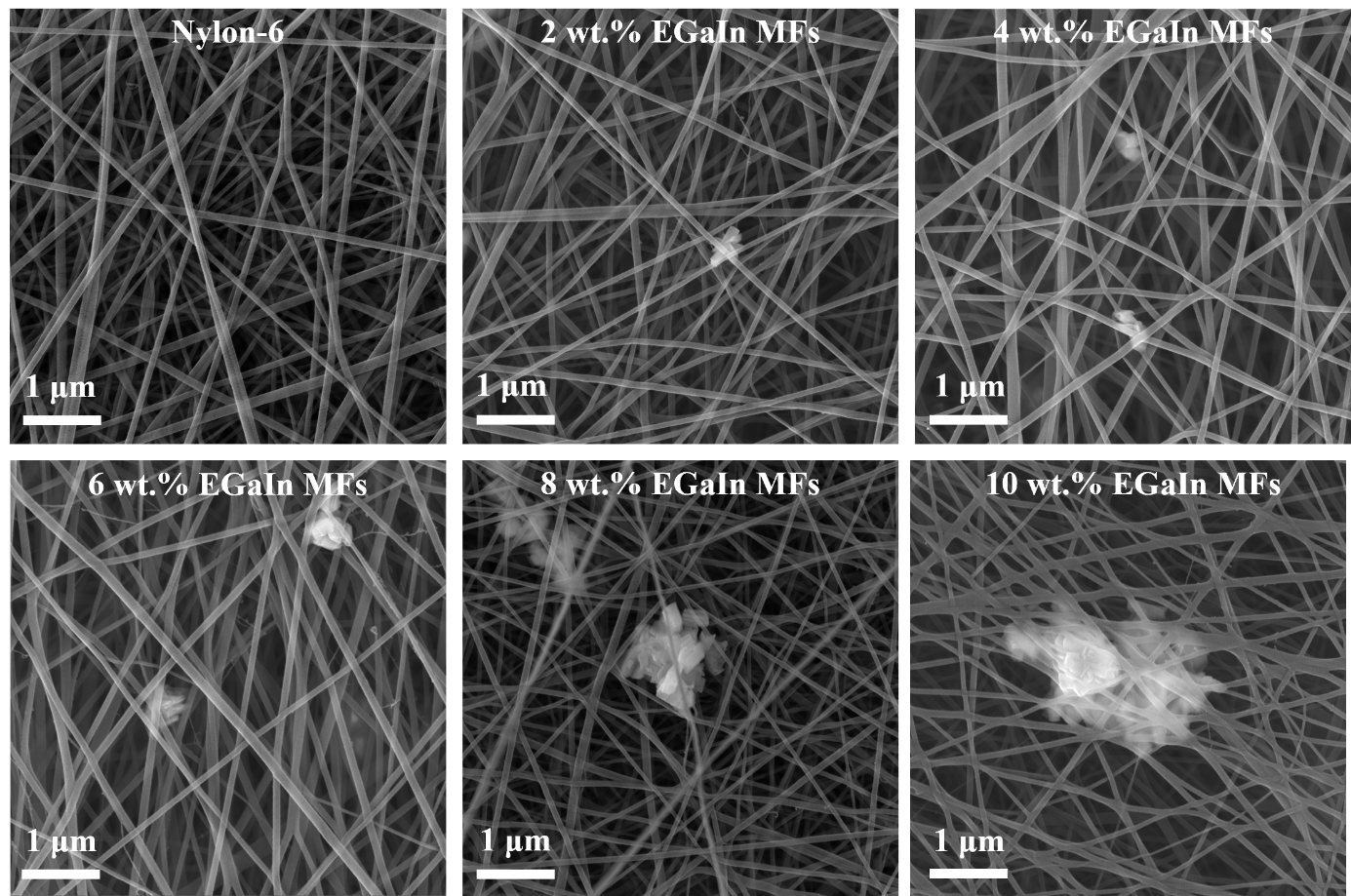


**Figure S3.** SEM images of Nylon-6-based nanofiber membranes with varying EGaIn MF contents.


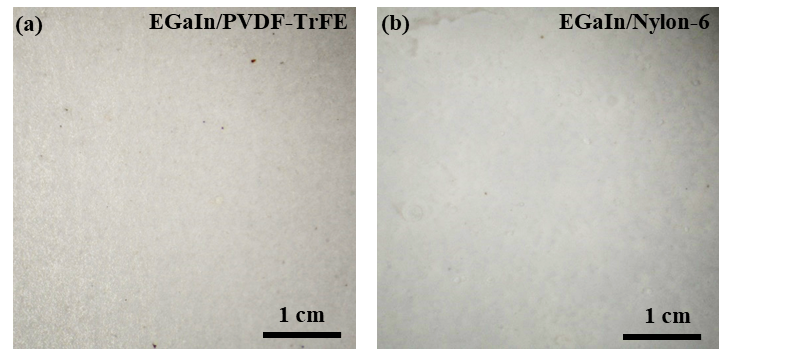


**Figure S4.** Optical microscopy images of nanofiber membranes containing 6 wt.% EGaIn MFs: (a) EGaIn/PVDF-TrFE and (b) EGaIn/Nylon-6.


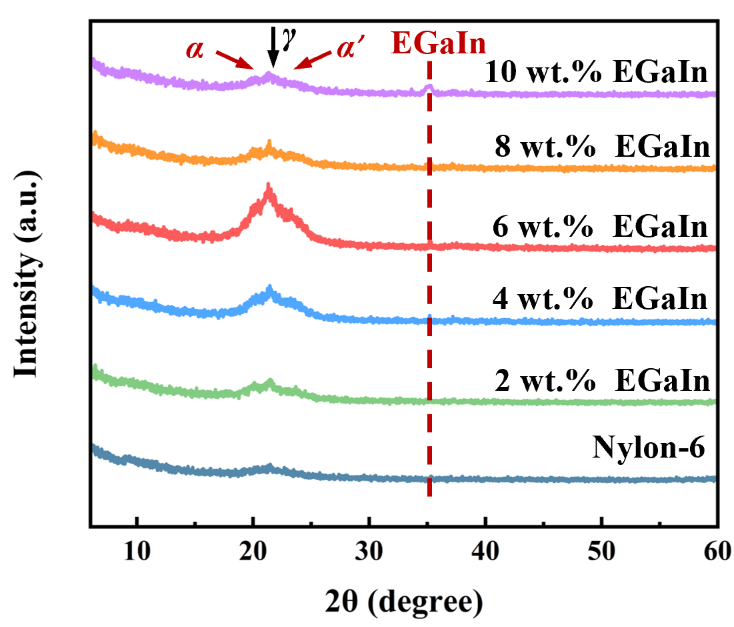


**Figure S5.** XRD analysis results of Nylon-6-based nanofiber membranes with varying EGaIn MF contents.


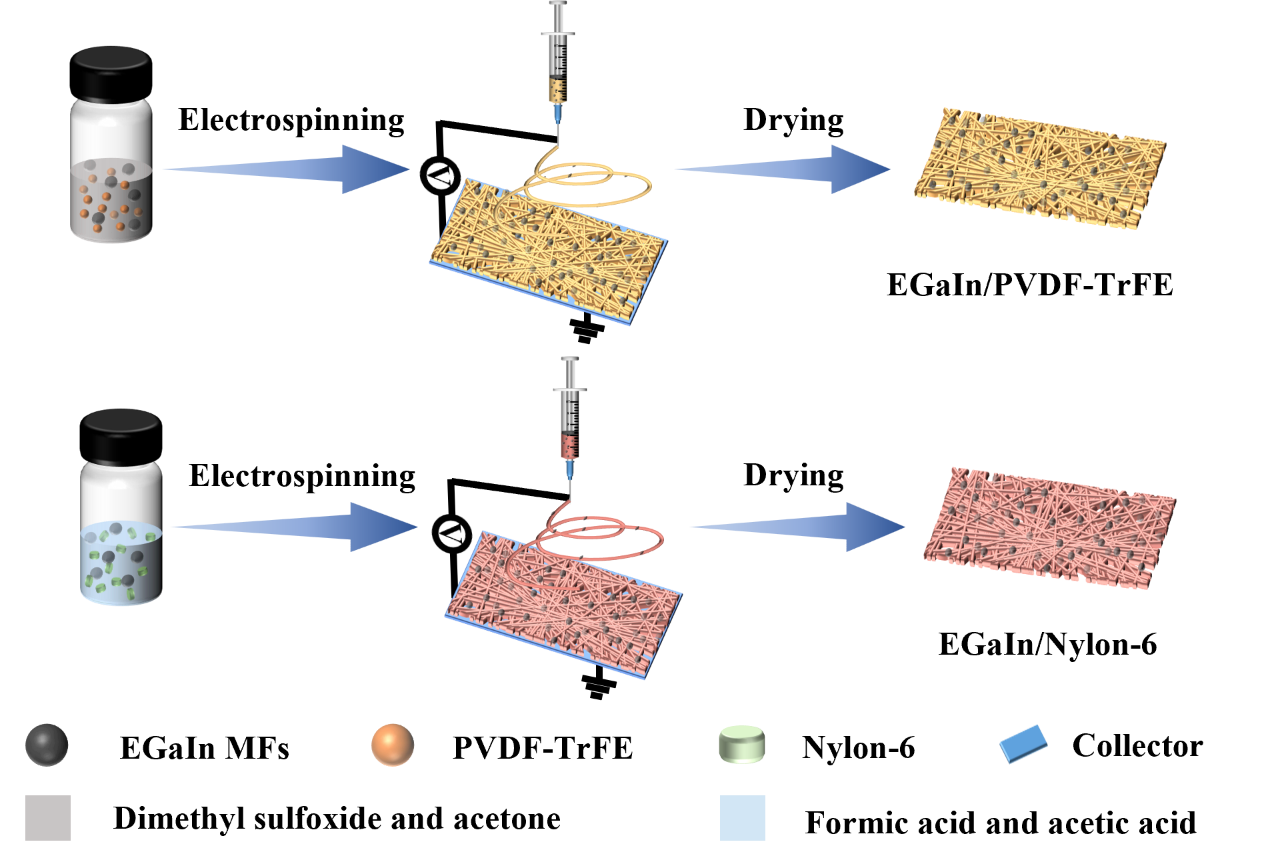


**Figure S6.** Schematic of the fabrication process of TENGs operating in the vertical contact-separation mode.


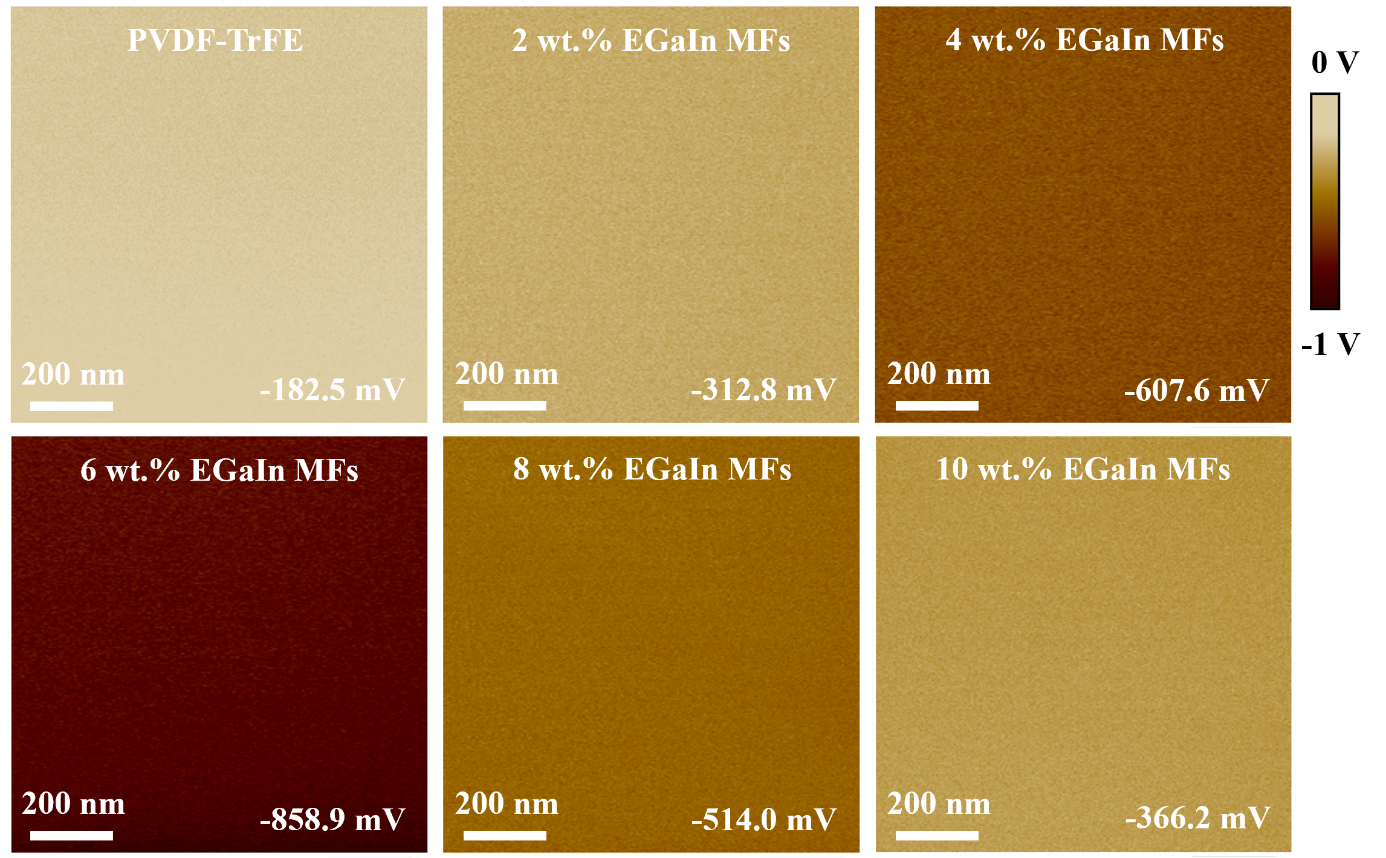


**Figure S7.** Surface potential of PVDF-TrFE-based nanofiber membranes with different EGaIn MF contents.


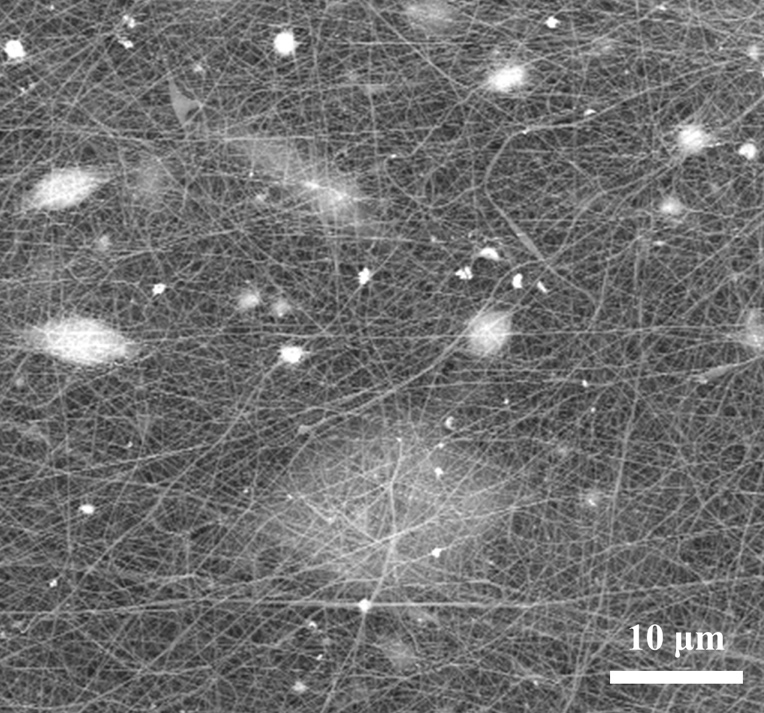


**Figure S8.** SEM image of the PVDF-TrFE nanofiber membrane with 10 wt.% EGaIn MFs.


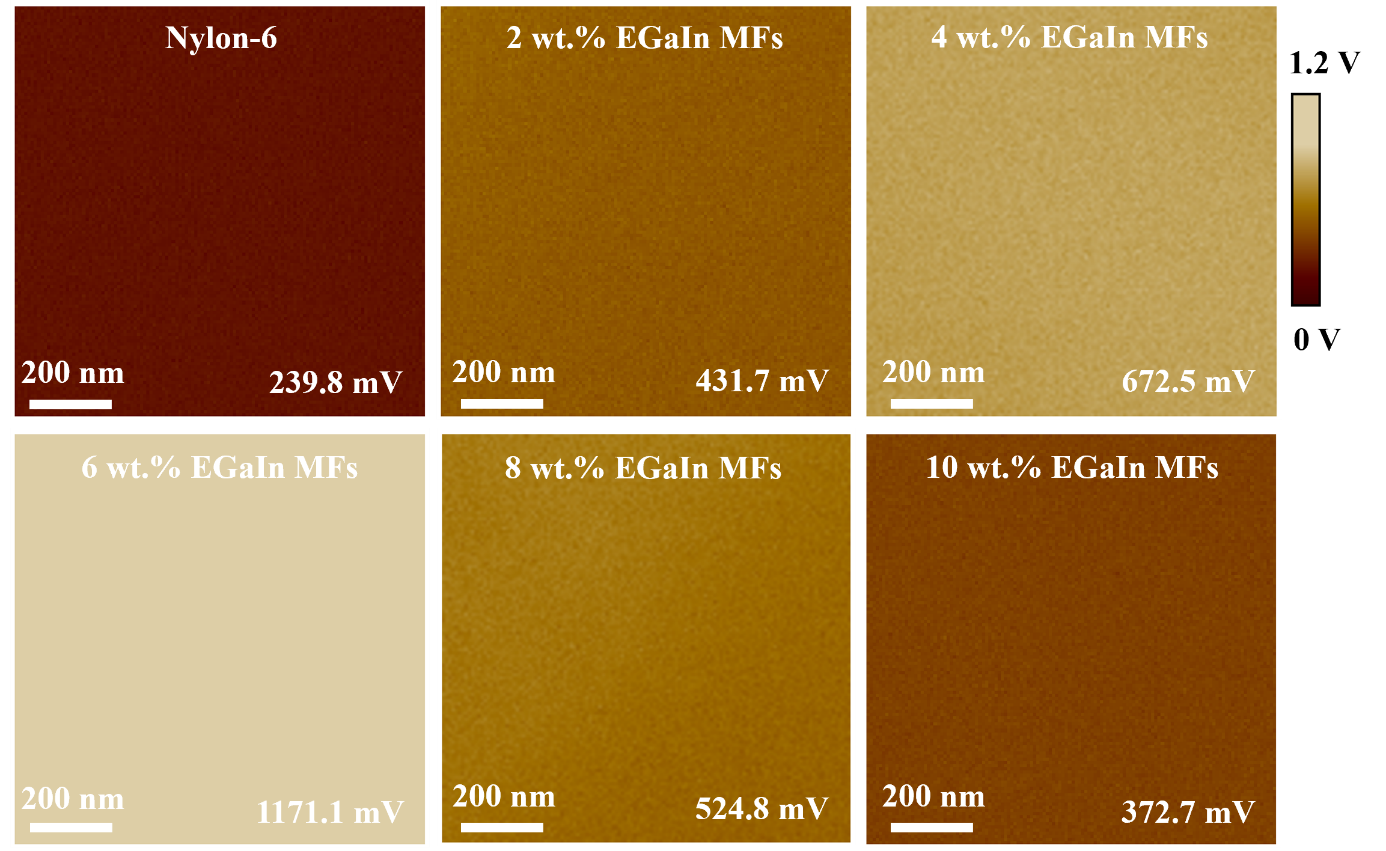


**Figure S9.**  Surface potential of Nylon-6-based nanofiber membranes with different EGaIn MF contents.


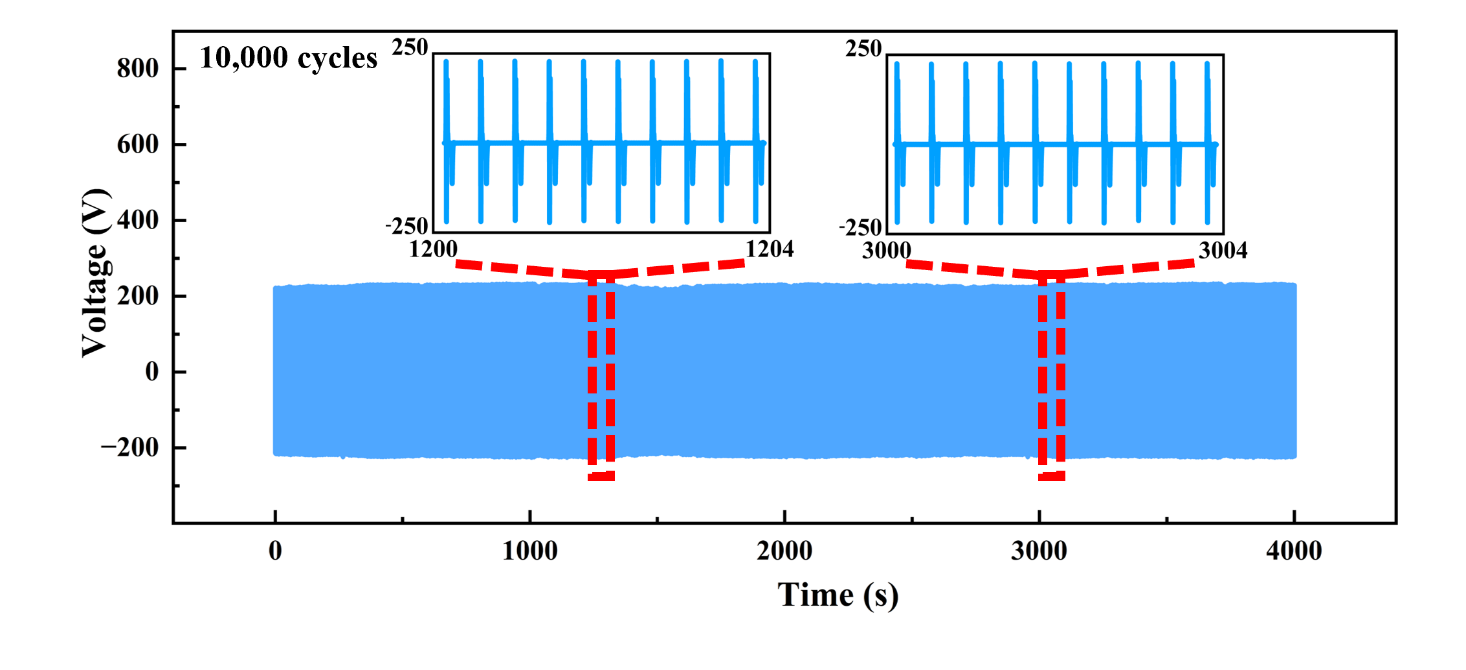


**Figure S10.** Stability of the optimized TENGs composed of 6 wt.% EGaIn/PVDF-TrFE and 6 wt.% EGaIn/Nylon-6 nanofibrous membranes over ~10,000 cycles at 2.5 Hz, subject to 60 N.


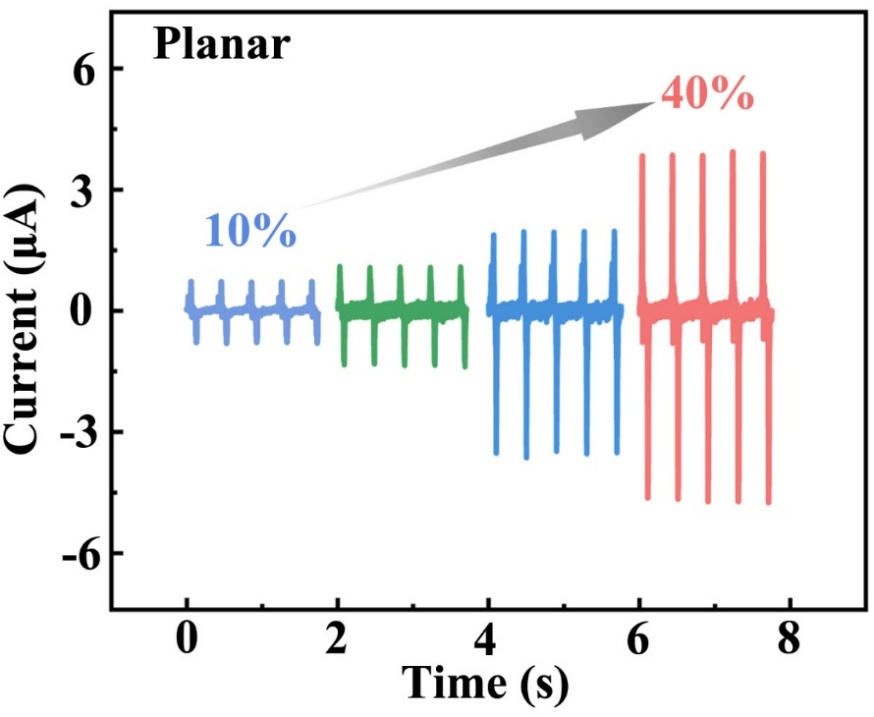


**Figure S11.** *I*_sc_ of the triboelectric devices with planar under tensile strains.


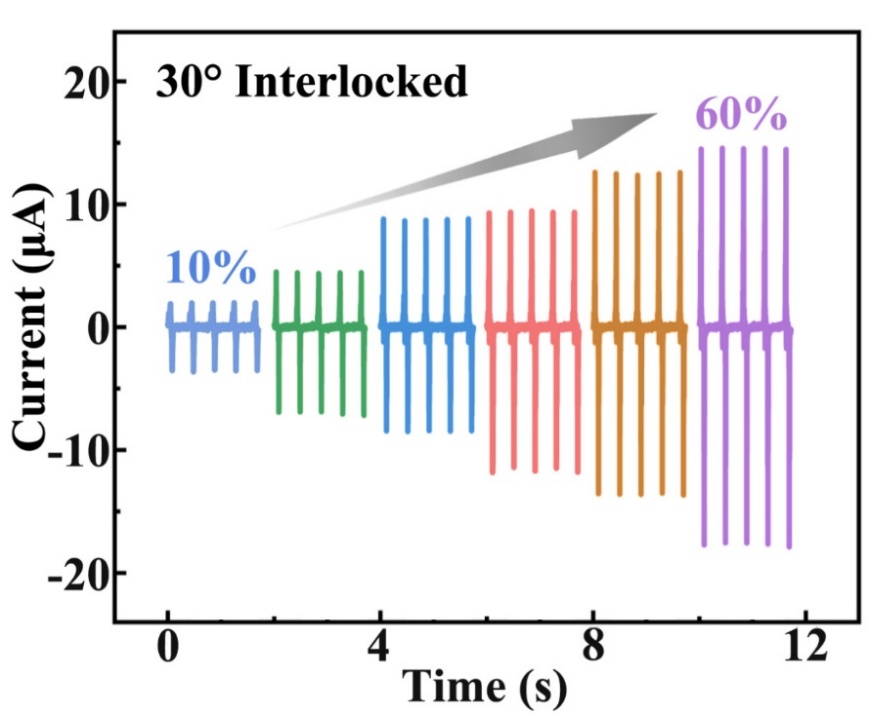


**Figure S12.** *I*_sc_ of the triboelectric devices with 30° interlocked under tensile strains.


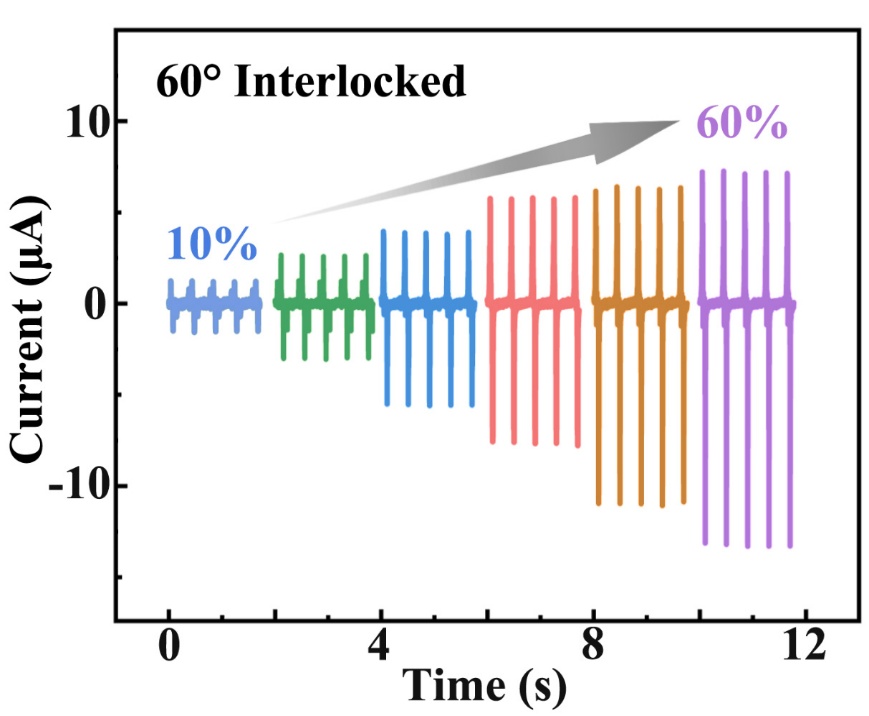


**Figure S13.** *I*_sc_ of the triboelectric devices with 60° interlocked under tensile strains.


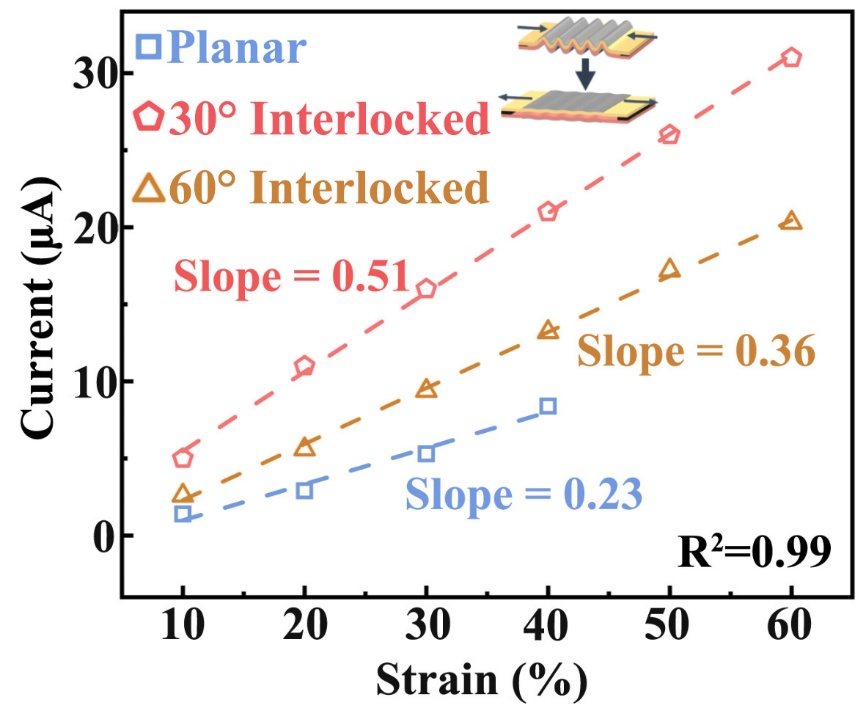


**Figure S14.** Current-strain curves of the interlocked stretchable TENGs with different configuration angles.


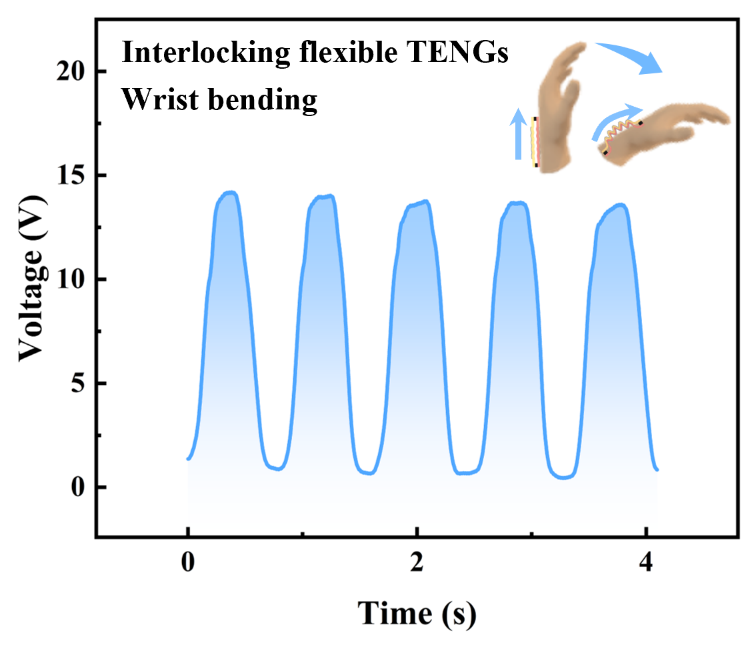


**Figure S15.** Electrical signals are generated by the interlocking flexible TENGs attached to wrists.


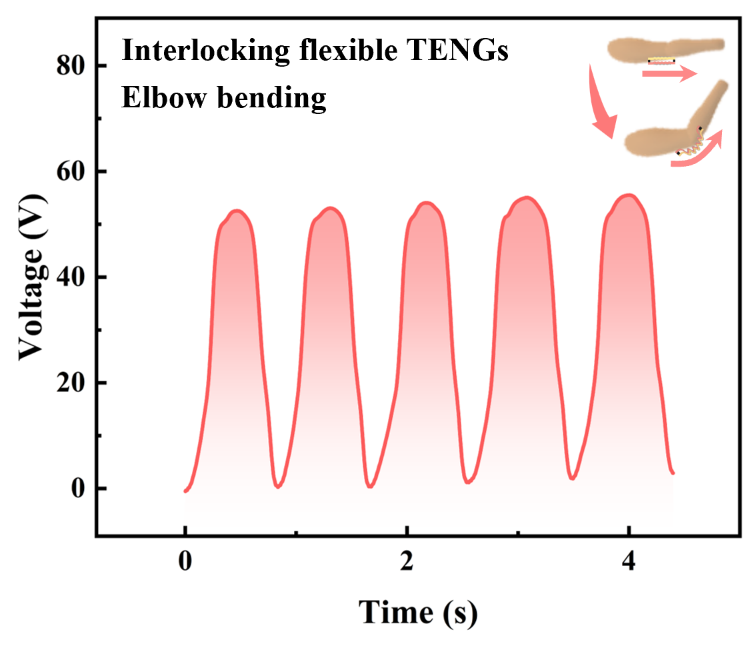


**Figure S16.** Electrical signals are generated by the interlocking flexible TENGs attached to elbows.

**Table S1.** Comparison of high-performance stretchable triboelectric nanogenerators.

| Material (s) | Voltage (V) | Current (μA) | Mechanical test cycles |
| --- | --- | --- | --- |
| ***This work*** | 188.6 | 31.1 | 10,000 |
| Lubricant/Ecoflex^1^ | 14.4 | 3.6 | 1000 |
| EGaIn/Ecoflex^2^ | 68.3 | - | 10,000 |
| alk-Ti_3_C_2_T_x_/PDMS@PTFE/PVA^3^ | 130.5 | - | 10,000 |
| EGaIn/CB/ExSil^4^ | 141.1 | 16.6 | - |
| Cu-EGaIn/PDMS^5^ | 91.0 | 13.3 | 4000 |

References

[1] C. Yang, Y. Wang, Y. Wang, Z. Wang, Y. Guo, L. Zhang, X. Liu, H. Chen, *Adv. Sci.* 2025, ***12***, e05363.

[2] L. Yang, L. Guo, Z. Wang, C. Meng, J. Wu, X. Chen, A. A. Musa, X. Jiang, H. Cheng, *Adv. Sci.* 2024, ***11***, e2405792.

[3] Y. Xiao, Q. Pu, C. Wang, X. Jia, S. Sun, Q. Jin, X. Wang, B. Wang, P. Sun, F. Liu, G. Lu, *Adv. Sci.* 2025, ***12***, e2416504.

[4] M. Singh, P. Bhuyan, S. Jeong, S. Park, *Adv. Funct. Mater.* 2024, ***35***, 2412178.

[5] J. Yang, J. Cao, J. Han, Y. Xiong, L. Luo, X. Dan, Y. Yang, L. Li, J. Sun, Q. Sun, *Nano Energy* 2022, ***101***, 107582.
